# The Use of Sea Buckthorn Processing Products in the Creation of a Functional Biologically Active Food Emulsion

**DOI:** 10.3390/foods11152226

**Published:** 2022-07-26

**Authors:** Lyubov V. Tereshchuk, Ksenia V. Starovoitova, Pavel A. Vyushinsky, Konstantin A. Zagorodnikov

**Affiliations:** 1 Department of Plant-Based Food Technology, Kemerovo State University, 650000 Kemerovo, Russia; tereshchuklubov@list.ru; 2 Zelenye Linii OOO, 143406 Krasnogorsk, Russia; vyushinskij@ssnab.ru; 3 Department of Customs and Commodity Expertise, Moscow State University of Food Production, 125080 Moscow, Russia; zagorodnikovka@mgupp.ru

**Keywords:** emulsions, emulsifiers, sea buckthorn, food additive, sea buckthorn oil, carotenoids, tocopherols, phospholipids, colloidal stability, oxidative stability

## Abstract

The current trend in dietary supplements and functional foods is the use of lipophilic bioactive compounds. The sea buckthorn (Hippóphae rhamnoídes) contains some such compounds: polyunsaturated fatty acids, tocopherols, and carotenoids. Lipophilic components are best distributed using oil-in-water emulsions, which ensures their high bioavailability. A significant property of emulsions is colloidal and oxidative stability, so the choice of emulsifiers that have both surface-active properties and antioxidant activity is an important area of research for making new types of food emulsions. The purpose of this study is the development and refinement of an emulsified biologically active food additive containing sea buckthorn products (pulp, juice, and oil) and stabilized with soy phospholipids. We studied the fruits of Chuyskaya, Orange, and Prevoskhodnaya sea buckthorn varieties growing in the Altai Territory. As we analyzed their composition, we chose the Chuyskaya variety for making the emulsion. The fruits contain 5.30 ± 0.1% of lipids including 16.8 ± 0.5 mg/100 g of carotenoids and 10.5 ± 0.5 mg/100 g of tocopherols. To choose the emulsifier we studied the fractional and fatty acid composition of the soy and sunflower phospholipids with different hydrophilic-lipophilic balances (HLB). We made the emulsions containing sea buckthorn oil and pulp of its different layers, soybean oil, and phospholipids by dispersion using an HG-15D homogenizer. The study of the colloidal stability showed that the most stable (99.5%) are the emulsions containing a mixture of hydrolyzed soybean phospholipids (HLB = 7) and fractionated soybean phospholipids (HLB = 3). The best ratio is 40:60. We examined the oxidative stability of the emulsions by provoking accelerated oxidation. The emulsions containing 1.5% of a soy phospholipids mixture showed the best oxidative stability. The resulting direct oil-in-water fine emulsion contains polyunsaturated fatty acids (PUFAs), tocopherols, β-carotene, and essential phospholipids. For this reason, the emulsion can be used to make biologically active food supplements (also encapsulated) and as part of special nutrients.

## 1. Introduction

It has been shown that nutrition directly affects the risk of lipid and carbohydrate metabolism disorders changing glucose and cholesterol levels, blood pressure, and body weight [1,2].

In terms of lipid metabolism disorder prevention, lipophilic biologically active substances including polyunsaturated fatty acids (PUFAs), phospholipids, fat-soluble vitamins, and antioxidant provitamins are of particular interest [3,4,5,6,7].

The sea buckthorn (Hippóphae rhamnoídes) is an organic product containing multiple lipophilic components [8,9,10,11]. The sea buckthorn exceeds many fruits, berries, and other plants in terms of the content of the biologically active substance. Sea buckthorn fruits contain water-soluble (C, PP, B1, B6, choline), fat-soluble vitamins, and provitamins (tocopherols, carotenoids, and vitamin K) [12]. Moreover, the content of carotenoids and tocopherols is unparalleled. The content of lipids in sea buckthorn fruits is from 3.4 to 12%. Moreover, 65–80% of the lipids are essential polyunsaturated acids. The sea buckthorn’s biologically active lipophilic components (tocopherols and carotenoids) are dissolved in the lipids of the fruits (sea buckthorn oil) [13,14].

Carotenoids and tocopherols can be produced from organic sources, including sea buckthorn fruits, to be used as nutraceuticals. There are several issues with their applications in foods and beverages since they are virtually insoluble in water. It usually means they should be dissolved in oils or dispersed in other suitable matrices before use in food [15,16,17].

Lipophilic components are best distributed as ultrafine oil-in-water emulsions with high bioavailability and biological efficacy compared to classical emulsions, where, due to the large size of the drops, it is difficult to protect the emulsion during the delivery [18,19,20,21,22,23].

The choice of the emulsifier is a major challenge in making food and pharmaceutical emulsions. It depends on the type of emulsion [24,25,26,27,28]. Surfactants with low hydrophilic-lipophilic balance (HLB) such as sorbitol monoesters are used to make water-oil emulsions. Surfactants with high HLB values are used to make oil-water emulsions [29,30]. In some cases, mixtures of surfactants with various HLB values are used as it improves emulsification [31].

Currently, organic surfactants are preferred. They are lactylated esters, glycerol and fatty acid esters, incomplete glycerides and derivatives, sucrose esters, polyoxyethylene sorbitan and fatty acid esters, and phospholipids (lecithin and lecithin derivatives) [32,33].

Phospholipids are amphiphilic molecules with a polar phosphate-based hydrophilic head and two non-polar hydrophobic fatty acid tails bound by a glycerol molecule. Both animal and plant phospholipids can contain many fractions depending on the substituent bound to the phosphorus acid group. The key phospholipids are phosphatidylcholine (PC), phosphatidylethanolamine (PE), phosphatidylserine (PS), phosphatidylinositol (PI), phosphatidic acid (PA), and phosphatidylglycerol. The most valuable fraction is phosphatidylcholine. Its molecules have both non-polar (two fatty acid groups) and polar (phosphorylcholine) parts [34]. It is this structure that accounts for the surfactant properties of phosphatidylcholine. It was also shown that the antioxidant activity of tocopherols can be improved by adding phosphatidylcholine (PC) and phosphatidylethanolamine (PE) [35,36,37,38]. Besides being a good emulsifier and antioxidant, phosphatidylcholine increases the bioavailability of accompanying nutrients and reduces cholesterol deposition in the liver by promoting phospholipid inhibition of cholesterol acyltransferase [39]. Various vegetable oils are a source of phospholipids. A study of the fractional composition of some plant phospholipids showed that soybean and sunflower oils have the highest content of phosphatidylcholines [39]. Plant phospholipids are extensively used in medications protecting the liver and in nutraceuticals and health-boosting foods [40,41,42].

The development of emulsified bioactive products and dietary supplements containing lipophilic ingredients requires more basic and applied research. Researchers and manufacturers should focus on the search for new, unconventional sources of target substances virtually not found in traditional oils. The production of lipophilic components from such sources is low-volume and requires a specific technology to process the raw material and its biologically active components [43].

The purpose of this study is the development and refinement of an emulsified biologically active food additive containing sea buckthorn products (pulp, juice, and oil) and stabilized with soy phospholipids. The study is structured as follows: First, an analysis of the composition and properties of the sea buckthorn fruits of different varieties cultivated in the Altai Territory is carried out to provide the rationale for selecting the sources of biologically active components; the composition and properties of the phospholipids obtained from different sources are studied; and finally, the effects of the added components on the final emulsion properties are also studied.

There are many studies on the composition and properties of sea buckthorn varieties [8,9,10,11,12,13,14,43] and plant phospholipids obtained from various sources [30,31,32,33,34,39]. However, the content of biologically active metabolites in plants depends not only on the species, but also on the variety, and local geographical and climatic conditions. In this regard, studying the composition and properties of phytochemical compounds in plants of different varieties of the same species is still a promising research area [44]. Furthermore, there are no prior studies of the food additive properties or foods with a composition identical to the one we proposed.

Our study will produce knowledge about obtaining biologically active components from organic sources and ways of adding them to dietary supplements and food products. The experimental results can be used in pharmaceutics and food production to make dietary supplements and health-boosting foods.

## 2. Materials and Methods

### 2.1. Materials

We studied the fruits of the Chuyskaya, Orange, and Prevoskhodnaya sea buckthorn varieties grown in the Altai Territory (2021 harvest) and purchased them at the local market. We also studied phospholipids purchased from Juvix-Pharm (Krasnodar, Russia). The manufacturer’s specifications listed the hydrophilic-lipophilic balance (HLB) of the emulsifiers. The following samples were selected: hydrolyzed soybean phospholipids (HLB 7); hydrolyzed sunflower phospholipids (HLB 8); fractionated soybean phospholipids (HLB 3); and fractionated sunflower phospholipids (HLB 2). The HLB for the simulated complex emulsifiers was determined by summing up the HLB values of the individual emulsifiers multiplied by the mass fraction of the emulsifier in the mixture.

All the laboratory kits and reagents used in the experimental research were purchased from Delta Origin (Moscow, Russia).

### 2.2. Sea Buckthorn Fruits Composition

In the first stage, we studied the composition and properties of the Chuyskaya, Orange, and Prevoskhodnaya varieties of sea buckthorn fruits grown in the Altai Territory. We studied the chemical composition of the juice, seeds, seed oil, and sea buckthorn pulp. We measured the moisture content, proteins, lipids, total sugar, vitamins, ash, acidity, and the physical and chemical parameters of the oil.

#### 2.2.1. Sea Buckthorn Fruits Crushing Procedure

We used an OM-350M-02F laboratory chopper (homogenizer) for grinding the sea buckthorn fruits (and to separate the seeds from pulp and shells). The unit consists of a drive; a mashing assembly; a paddle + a 3 mm mashing disk. Its rated mashing performance is 400 kg/h, the cell diameter is 3 mm, and the rpm is 300 ± 10.

The unit fine grinds the shell and pulp while the seeds do not pass through the gap and remain intact. The resulting crushed sea buckthorn pulp is separated into three layers when stored for 6 h:

− The bottom layer is a clarified juice (65%)
− The middle layer is the middle layer pulp (30%)
− The top layer contains the greatest amount of lipids (5%).

To analyze the chemical composition of the layers the separated pulp was frozen at −18 °C and then cut with a knife.

#### 2.2.2. Spectrophotometric Studies of the Sea Buckthorn Fruits

The mass fractions of the dry substances, reducing sugars, and moisture in the pulp layers was determined by near-IR spectroscopy with a MATRIX IR spectrometer from Bruker Optics (Billerica, MA, USA).

#### 2.2.3. Atomic Emission Analysis of the Sea Buckthorn Fruits

We determined the content of macro- and microelements in the sea buckthorn fruits (such as P, Na, Ca, K, Mg, Fe, Ti, and Al) by atomic emission analysis using a Plasma 1000 instrument from Perkin Elmer (Waltham, MA, USA). The instrument is equipped with an automatic sampler and is computer controlled.

### 2.3. Sea Buckthorn Oil Composition Analysis

#### 2.3.1. Sea Buckthorn Oil Making

We made sea buckthorn oil from the dried and crushed pulp and seeds by subcritical CO_2_ extraction on a pilot extraction unit with a five-liter stainless steel vessel. The extraction conditions were 28 °C, 7 MPa, and 120 min, respectively; high-purity (99.999%) carbon dioxide was used as a solvent. We decided to apply this extraction method since it gently treats the thermolabile compounds in sea buckthorn.

#### 2.3.2. Physical and Chemical Studies of the Sea Buckthorn Oil

We measured the density of the sea buckthorn oil samples obtained from the pulp and seeds with an areometer. The instrument was dipped into the oil and the reading was recorded.

The iodine number of the sea buckthorn oil was measured with the Wijs method. It uses a solution of iodine monochloride in glacial acetic acid as a halogen reagent.

The acid number of the sea buckthorn oil which indicates the content of free fatty acids was determined by dissolving lipids in an ether-alcohol mixture (2:1), followed by rapid titration of the sample with a sodium hydroxide solution in the presence of a phenolphthalein pH indicator until a weak pink coloration was achieved.

The peroxide number of the sea buckthorn oil was measured by analyzing the reaction of the lipid oxidation products (peroxides and hydroperoxides) with potassium iodide in acetic acid and chloroform, followed by assaying of sodium thiosulfate.

#### 2.3.3. Gas-Liquid Chromatography

The composition of the fatty acids and phospholipids in the lipid phase of the samples was studied by gas-liquid chromatography. A chromatogram of the fatty acid methyl esters of the sample’s lipid phase is made followed by the identification and assaying of the components by peak area. Each component of the fatty acid methyl ester mixture is characterized by a specific retention time (from the sample introduction to the peak on the chromatogram). An Agilent 7890A gas chromatograph was used. The Polychrome software processed the results.

#### 2.3.4. Spectrophotometric Studies of the Sea Buckthorn Oil

The total content and fractional composition of tocopherols in the sea buckthorn oil, vitamin content, and the content of carotenoids and flavonoids were identified by IR spectrometry with a MATRIX IR spectrometer from Bruker Optics, Bruker (Billerica, MA, USA).

### 2.4. Preparation of Sample Emulsions

Direct emulsions (oil in water) (100 g each) were made in a lab environment. The aqueous phase consisted of sea buckthorn juice and middle layer pulp (53%). The fat phase consisted of sea buckthorn oils derived from the pulp and the seeds mixed with soybean oil and phospholipids (47%). The emulsions were prepared in stages. The aqueous phase was made from the clarified juice and middle layer pulp by magnetic stirring and pasteurization at 60 °C for 30 min. The pasteurization temperature was chosen to account for the high thermolability of carotenoids and ascorbic acid contained in the sea buckthorn pulp. Concurrently, the emulsifier was prepared for introduction into the fat phase. The soybean phospholipids were dissolved in refined deodorized soybean oil and heated to the phospholipid granule’s complete melting temperature (65 °C). Then, the aqueous phase was cooled to 20 °C to make a coarse emulsion by gradual addition of the sea buckthorn seed oil, pulp oil, and emulsifier dissolved in soybean oil while stirring with a lab stirrer. Then, the emulsion was homogenized by an HG-15D homogenizer (DAIHAN Scientific, Seoul, Korea) at 15,000 rpm. Three emulsions were prepared with various phospholipid content (1.0 wt%; 1.5 wt% and 2.0 wt%). The mass fraction of phospholipids was controlled by reducing the sea buckthorn oil amount. The final emulsions were stored in a refrigerator at 6 ± 0.5 °C for 24 h before other studies.

### 2.5. Sample Emulsions Properties

#### 2.5.1. Colloid and Thermal Stability of the Emulsions

To determine the colloidal and thermal stability of the emulsions, we filled lab tubes with the emulsion samples to the top mark and centrifuged the tubes for 5 min at 1500 rpm. Then, the tubes were placed in a boiling water container. The water level was equal to the level of the samples in the tubes. The tubes were kept there for 3 min while preventing the water from intense boiling and then centrifuged again for 5 min at 1500 rpm. Then, the volume of the intact emulsion was measured by the scale on the tube with a separated aqueous or fat phase. The emulsion stability as a percentage of the intact emulsion by volume was estimated as

𝑋 = 𝑉∙10010,

where *V* is the volume of intact emulsion, cm^3^; 10 is the sample volume, cm^3^.

#### 2.5.2. Oxidative and Hydrolytic Stability of the Emulsions

The oxidative and hydrolytic stability of the emulsions under accelerated oxidation was studied by estimating the peroxide and acid numbers of the lipid phase separated from the emulsion. The emulsion samples were stored for 14 days in a transparent glass container with free access to oxygen and light and at 25 °C. Samples were taken every day.

The lipid phase of the emulsion not mixed with the aqueous phase and not containing phospholipids was the reference sample. The following samples were prepared:

− Sample 1: Lipid phase of emulsion before emulsification (reference);
− Sample 2: Lipid phase of the emulsion containing 1.0% of phospholipids;
− Sample 3: Lipid phase of the emulsion containing 1.5% of phospholipids;
− Sample 4: Lipid phase of the emulsion containing 2.0% of phospholipids.

After the storage and accelerated oxidation, the fat phase was extracted as follows: a 5 g emulsion sample was placed in a 250 cm^3^ conical flask with a ground joint, and 15 cm^3^ of ethyl alcohol was gradually added to the sample and stirred with a glass rod until a suspension was obtained. Then, 30 cm^3^ of chloroform was added to the flask while stirring. An HCN-300-29/32 glass spiral refrigerator (made in Russia) was used to cool it down. The mixture was then boiled in a water bath for 1 h while stirring in a gentle circular motion. After, the sample was cooled to room temperature. Then, the sample was filtered through a glass funnel with a filter pre-moistened with the extraction mixture. Solvents were removed from the extract first by evaporation in a water bath, then in a desiccator for 30 min at (105 ± 5) °C. The crucible with the fat phase was cooled at room temperature for (20 ± 5) min and weighed to measure the mass fraction of fat.

Peroxidation of the emulsion lipid phase was estimated using the reaction of lipid oxidation products (peroxides and hydroperoxides) with potassium iodide in acetic acid and chloroform, followed by assaying the sodium thiosulfate.

The acid number of the emulsion lipid phase was estimated by dissolving the lipids in an ether-alcohol mixture (2:1), followed by rapid titration of the sample with sodium hydroxide solution in the presence of a phenolphthalein indicator until a weak pink color was reached.

### 2.6. Statistical Analysis

All the studies were repeated three times. The results were processed with the standard mathematical statistics methods and the Statistica 10.0 software (StatSoft Inc., 2007, Tulsa, OK, USA). Variance in the phytochemicals content was analyzed by the use of ANOVA with Tukey’s test for homogenous groups. The differences between the mean values were considered significant when the confidence interval was less than 5% (*p* ≤ 0.05).

## 3. Results and Discussion

### 3.1. Composition of the Sea Buckthorn Fruits

We experimentally studied the composition of the sea buckthorn fruits grown in the Altai Territory. The results are listed in Table 1.

Table 2 lists the contents of some bioactive lipophilic and water-soluble substances of the sea buckthorn varieties.

The study detected significant differences in the chemical composition of the Chuyskaya, Orange, and Prevoskhodnaya sea buckthorn (Hippóphae rhamnoídes) varieties grown in the Altai Territory. In general, the result agrees with the data obtained by Olas, B. (2016) [9,11] Teleszko, M. et al. (2015) [12], and Kumar, R. et al., (2011) [13]. Specifically, Teleszko, M. et al. (2015) [12] reported the carotenoid content in sea buckthorn between 6.19 and 23.91 mg/100 g fresh weight (*p* < 0.05). The analysis identified the Chuyskaya variety as the one with the highest content of dry matter (14.5 ± 0.2%) and lipids (5.30 ± 0.1%), including carotenoids (16.8 ± 0.5 mg/100 g) and tocopherols (10.5 ± 0.5 mg/100 g). Furthermore, out of the studied varieties, Chuyskaya has a higher content of ascorbic acid and polyphenolic substances. The differences between the varieties in the content of the target substances cannot be called significant. However, the Chuiskaya variety still demonstrated a slight superiority in all respects. For further research, this particular variety was chosen. In addition, we were guided by the greatest availability and prevalence of this variety of sea buckthorn in the region.

### 3.2. Properties of the Fractions Obtained by Crushing the Sea Buckthorn Fruits of the Chuiskaya Variety

At the next stage, we investigated the products obtained by crushing the sea buckthorn fruits of the Chuiskaya variety.

After removing the seeds, the sea buckthorn pulp was poured into PET bottles for storage. After 6 h, the pulp formed three layers: clarified juice, bottom pulp, and top pulp. Table 3 lists the chemical composition of the sea buckthorn juice and pulp.

Table 3 shows that clarified sea buckthorn juice contains almost no lipids, tocopherols, or carotenoids, but contains a large amount of ascorbic acid (204.80 ± 1.2 mg/100 g). As the study shows, the clarified berry juice composition is as follows: mass fraction (%) of moisture (94.40 ± 0.5); solids (5.6 ± 0.5) including total sugar (3.20 ± 0.1), protein (0.06 ± 0.01), organic acids (1.8 ± 0.1), ash (0.04 ± 0.01). The juice density is 1038 kg/m^3^.

The chemical composition of sea buckthorn seeds was also investigated. The mass fraction of moisture was 11.0 ± 1.0%, nitrogenous substances: 8.0 ± 0.5%, oil: 10.0 ± 1.0%, ash: 2.0 ± 0.2%, fiber: 20.0 ± 2.0%.

### 3.3. Properties of the Sea Buckthorn Lipids

Table 4 lists the physical and chemical properties of the lipids from the seeds and pulp of the Chuyskaya sea buckthorn variety.

The lipid fraction isolated from the pulp contained 320 mg of carotenoids per 100 g and 90 mg of tocopherols per 100 g. The carotenoids composition study identified α, β, γ-carotenoids, lycopene, poly-cis-lycopene-3, and zeaxatin. The content of the most physiologically active β-carotene in the fruits was 45% of the total amount of carotenoids. Buckthorn tocopherols are overwhelmingly represented by the most physiologically active α-tocopherols (57% of the total amount of tocopherols). γ- and δ-tocopherols were also found.

The chemical composition of the seed oil differs significantly from the oil obtained from the pulp in terms of fatty acid composition and the content of the key lipophilic components. For instance, 11.1 ± 0.05 mg/100 g of carotenoids and 160.0 ± 0.1 mg/100 g of tocopherols were found in the oil obtained from the seeds by subcritical extraction. These results agree with the data provided by Li Zheng et al. (2017), who investigated the composition of sea buckthorn oil obtained by subcritical extraction. They found that the seed oil had a carotenoid content of 15.1 ± 0.1 mg/100 g, the oil obtained from the pulp had 104.6 ± 2.0 mg/100 g, while the tocopherol content was 245 ± 1 mg/100 g for the seed oil and 198 ± 1 mg/100 g for the pulp oil [8].

### 3.4. Emulsifier Selection and Rationale

To make a food additive as an emulsion, we used sea buckthorn pulp, sea buckthorn juice, sea buckthorn oil obtained from the pulp as a source of carotenoids, and seed oil as a source of tocopherols. We also used surfactants with relatively low hydrophilic-lipophilic balance suitable for stabilizing oil-water emulsions.

A significant property of emulsions is colloid and oxidative stability and the ability to preserve and deliver biologically active components. The development of emulsifiers that have both surfactant properties and biological efficacy is an important area of research for making new fine, biologically active emulsions.

To select the most suitable surfactant with the required processing properties and biological activity, we investigated some samples of soybean phospholipids (SBP) and sunflower phospholipids (SFP) with different hydrophilic-lipophilic balances, widely used in food and pharmaceutical emulsion production.

The fractional composition of phospholipids was investigated (Table 5).

As reported in the available sources, the surface activity of lecithin is due to the presence of a phosphatidylcholine fraction. Its molecule has non-polar (two fatty acid groups) and polar (phosphorylcholine) parts [32,34]. We found that the examined phospholipid samples contain 45% or more of this fraction.

Next, the fatty acid composition of soybean and sunflower phospholipids was examined (Table 6).

The study of the fractional and fatty acid composition of soy and sunflower phospholipids confirms the data from such researchers as Caysen A. et al., (2022) [34].

It is feasible to add soy phospholipids to the emulsion thanks to the presence of 1,2-dilinoleoyl-phosphatidylcholine, which is not synthesized by the human body. The advantage of this special phospholipid form compared to endogenous ones is the presence of two essential polyunsaturated fatty acids as glyceride compositions. It facilitates easy incorporation into hepatocytes when consumed, and the restoration of the protective cell wall phospholipid layer protective properties [37,39,40].

Two samples of soy phospholipids were selected for further studies: hydrolyzed soy phospholipids (HLB 7) and fractionated soy phospholipids (HLB 3).

### 3.5. Colloidal Stability of the Emulsions

The reference oil-in-water emulsions containing sea buckthorn pulp, oil pulp, and sea buckthorn seed oil mixed with soybean oil and phospholipids were made in a laboratory environment. First, a coarse emulsion was obtained with a lab stirrer; then the emulsion was homogenized with an HG-15D lab homogenizer. Two types of soybean phospholipids in different amounts as well as a mixture of phospholipids with different HLB values served as surfactants. The colloidal stability of the resulting emulsions was estimated (Table 7).

The study showed that the emulsions made with a mixture of hydrolyzed soybean phospholipids (HLB = 7.40%) and fractionated soybean phospholipids (HLB = 3.60%) have the best colloidal stability. The hydrophilic-lipophilic balance of the resulting complex emulsifier is 4.6, an optimal value for stabilizing oil-in-water emulsions.

### 3.6. Oxidative and Hydrolytic Stability of the Emulsions

**Figure 1:**
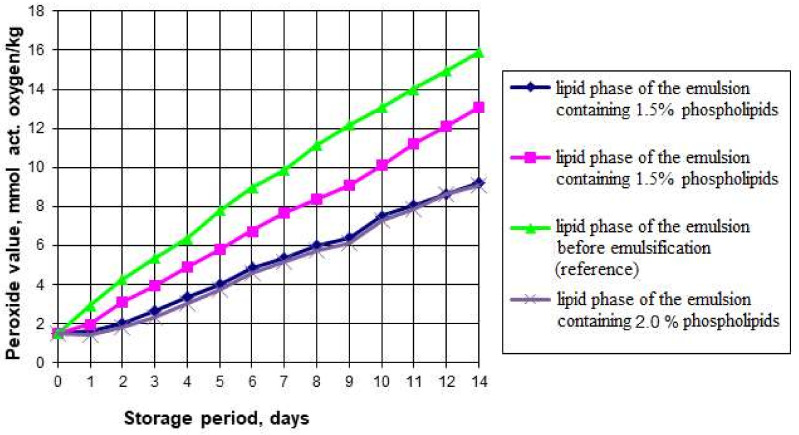
Peroxide number in the phospholipid emulsions during accelerated oxidation.

**Figure 2:**
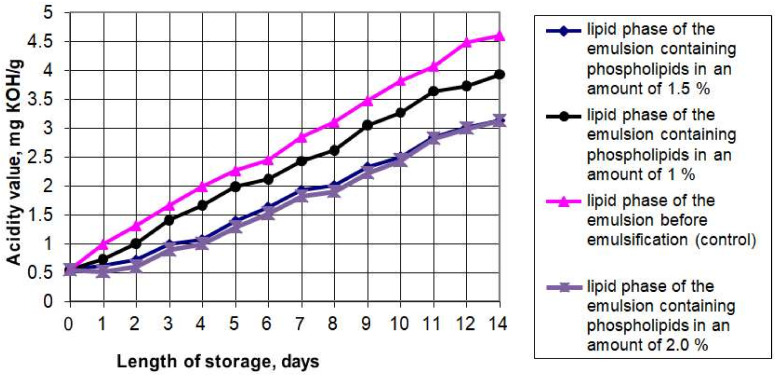
The acid number of the phospholipid emulsions during accelerated oxidation.

The presence of the aqueous phase plus polyunsaturated fatty acids of the fat phase can provoke oxidative and hydrolytic processes during the emulsion storage, resulting in the breakdown of the fatty acids, oxidation of the triglycerides and isomerization of the original acids. Accumulation of such oxidation products as peroxides, hydroperoxides, aldehydes, ketones, and oxy compounds can contribute to the deterioration of organoleptic and rheological properties and reduce its physiological and biological value. For this reason, we studied the oxidative and hydrolytic stability of the emulsions and the effects of the phospholipid content on these properties. Figure 1 and Figure 2 show the graphs of the accelerated oxidation and hydrolysis of the emulsions during their storage for 14 days in a transparent glass container with free access to oxygen and light at 25 °C.

The graphs show that the peroxide number (PV) of the emulsion lipid phase without phospholipids increased from 1.5 to 15.9 mEqO_2_/kg. The peroxide number of the emulsion’s fat phase with 1% phospholipids added increased from 1.5 to 13.1 mEqO_2_/kg in two weeks of storage under accelerated oxidation conditions. The samples containing 1.5% and 2% of phospholipids showed the highest oxidative stability: their peroxide number increased from 1.5 to 9.2 mEqO_2_/kg. A study of the acid number (AV) variation showed that the lowest acid number at the end of the accelerated oxidation period was also found in the samples containing 1.5% and 2% of phospholipids. Since increasing the mass fraction of phospholipids in the emulsions had no significant effect on the oxidative and hydrolytic deterioration change rate, we concluded that increasing the mass fraction of phospholipids in emulsions to 2% is inexpedient.

The results also confirmed that soy phospholipids have antioxidant properties and can slow down the oxidation process in polyunsaturated fatty acids, which predominate in the fat phase of the emulsion. The available research literature presents studies on the combined effect of tocopherols and phospholipids on the oxidative stability in lipids. The data we obtained do not contradict the results presented by such researchers as McClements DJ and Decker E. (2018) [35], Xu N et al. (2019) [36], and Kwon Y et al. (2019) [37]. For instance, Samdani GK et al. (2018) [38] showed that phospholipids create synergy with tocopherols and inhibit lipid oxidation in oil-in-water emulsions. It can be a new antioxidant clean label strategy for food emulsions.

### 3.7. Emulsion Quality Factors

We investigated the quality factors of the emulsions obtained in a laboratory environment. The results are presented in Table 8.

The oil-in-water emulsion we made contains polyunsaturated fatty acids (PUFAs), tocopherols, carotenoids, and essential phospholipids. With such content, the emulsion is suitable for the production of biologically active food additives, also encapsulated, and health-boosting foods.

## 4. Conclusions and Recommendations

As a result of this study, a food emulsion containing pulp, juice, pulp oil, and oil of sea buckthorn seeds stabilized with soy phospholipids was obtained. After analyzing the chemical composition of the fruits of Chuyskaya, Orange and Prevoskhodnaya sea buckthorn varieties grown in the Altai Territory, the Chuyskaya variety was selected for making the emulsion. The variety of fruits contains 5.30 ± 0.1% of lipids, including 16.8 ± 0.5 mg/100 g of carotenoids and 10.5 ± 0.5 mg/100 g of tocopherols. To choose the emulsifier, we studied the fractional and fatty acid composition of the soy and sunflower phospholipids with different hydrophilic-lipophilic balances (HLB). This study has some restrictions since the design and methods were chosen to match the specific chemical composition of certain species and varieties. It is assumed that the content of bioactive compounds in sea buckthorn samples of other varieties, or Chuyskaya, Orange, and Prevoskhodnaya varieties harvested at a different time and location, and phospholipids obtained from other sources, may have a significantly different chemical composition which requires making changes to the application of such components in foods and food supplements. The study of emulsion colloidal stability showed that the most stable (99.5%) emulsions contain a mixture of hydrolyzed soybean phospholipids (HLB = 7) and fractionated soybean phospholipids (HLB = 3) in the 40:60 ratio. By analyzing the fractional and fatty-acid composition of phospholipids, we chose the most suitable sample of soy phospholipids containing 1,2-dilinoleoyl-phosphatidylcholine not synthesized by the human body to be added to the emulsion. The studies of the emulsion oxidative stability with various phospholipid contents also confirmed that the highest antioxidant activity of the substance is achieved at 1.5% wt. concentration. The study showed that a further increase in the phospholipid content to 2% does not affect the oxidative and hydrolytic stability of the emulsions.

The resulting direct oil-in-water fine emulsion contains polyunsaturated fatty acids (PUFAs), tocopherols, β-carotene, and essential phospholipids. For this reason, the emulsion can be used to make biologically active food supplements (also encapsulated) and as part of special nutrients.

The study results will be used in further research to develop efficient methods of adding plant lipophilic components to food systems for making food, beverages, and pharmaceutical products.

The use of processed sea buckthorn and plant phospholipids for making therapeutic, health-boosting foods and dietary supplements will increase the nutritional and therapeutic properties. Regular taking such products will reduce the risk of metabolic diseases.

## Figures and Tables

**Table 1:** Chemical composition of sea buckthorn fruits.

	Mass Fraction %					
Variety	Dry Substances	Titratable Acids (Expressed as Malic Acid)	Total Sugar	Ashes	Protein	Lipids
Prevoskhodnaya	13.5 ± 0.2 ^b^	2.04 ± 0.14 ^c^	4.48 ± 0.12 ^d^	0.38 ± 0.02 ^b^	1.90 ± 0.02 ^c^	4.73 ± 0.09 ^a^
Orange	13.9 ± 0.2 ^a^	2.54 ± 0.15 ^b^	3.23 ± 0.18 ^c^	0.43 ± 0.02 ^b^	1.88 ± 0.02 ^c^	5.06 ± 0.11 ^a^
Chuyskaya	14.5 ± 0.2 ^a^	2.26 ± 0.12 ^a^	4.72 ± 0.16 ^d^	0.32 ± 0.02 ^a^	1.92 ± 0.02 ^c^	5.30 ± 0.12 ^c^

^a^, ^b^, ^c^, ^d^—Means in the same line for all cultivars followed by different letters are significantly different (*p* ≤ 0.05). Values are mean ± SD (*n* = 3). SD—Standard deviation.

**Table 2:** Vitamins in the sea buckthorn fruits.

Vitamins	Content, mg/100 g		
	Chuyskaya	Orange	Prevoskhodnaya
Carotenoids, total	16.8 ± 0.3 ^d^	15.5 ± 0.3 ^a^	15.0 ± 0.3 ^b^
β-carotene	5.5 ± 0.1 ^d^	5.0 ± 0.1 ^a^	4.8 ± 0.1 ^b^
Tocopherols, total	10.5 ± 0.3 ^c^	9.3 ± 0.3 ^b^	9.0 ± 0.3 ^b^
Vitamin C	172 ± 2 ^c^	168 ± 2 ^d^	170 ± 2 ^d^
Polyphenols	150 ± 2 ^d^	143 ± 2 ^d^	147 ± 1 ^c^

^a^, ^b^, ^c^, ^d^—Means in the same line for all cultivars followed by different letters are significantly different (*p* ≤ 0.05). Values are mean ± SD (*n* = 3). SD—Standard deviation.

**Table 3:** Chemical composition of the sea buckthorn pulp.

Property	Sea Buckthorn Pulp Components		
	Clarified Juice	Top Layer Pulp	Bottom Layer Pulp
Moisture mass fraction, %	94.40 ± 0.3 ^d^	76.8 ± 0.40 ^a^	79.20 ± 0.3 ^b^
Dry matter, % incl.	5.6 ± 0.3 ^d^	23.2 ± 0.4 ^b^	20.8 ± 0.3 ^c^
lipids, %	1.1 ± 0.10 ^d^	10.75 ± 0.30 ^b^	7.15 ± 0.21 ^c^
protein, %	0.06 ± 0.01 ^c^	1.48 ± 0.03 ^d^	1.66 ± 0.03 ^d^
fiber, %	0.05 ± 0.01 ^c^	3.90 ± 0.10 ^d^	4.30 ± 0.10 ^d^
total sugars, %	3.20 ± 0.10	4.20 ± 0.10 ^a^	4.60 ± 0.10 ^b^
ash, %	0.04 ± 0.01 ^a^	0.24 ± 0.02 ^a^	0.36 ± 0.02 ^a^
titratable acids, mg/100 g	1.80 ± 0.10 ^d^	2.50 ± 0.04 ^d^	2.60 ± 0.05 ^b^
carotenoids, mg/100 g	-	27.77 ± 0.15 ^d^	30.52 ± 0.16 ^b^
tocopherols, mg/100 g	-	17.80 ± 0.18 ^d^	18.90 ± 0.20 ^b^
flavonoids, mg/100 g	10.20 ± 0.20 ^c^	42.70 ± 0.45 ^b^	67.50 ± 0.53 ^c^
Ascorbic acid, mg/100 g	204.80 ± 4.8 ^c^	61.70 ± 1.1 ^b^	83.70 ± 1.5 ^c^

^a^, ^b^, ^c^, ^d^—Means in the same line for all pulp components followed by different letters are significantly different (*p* ≤ 0.05). Values are mean ± SD (*n* = 3).

**Table 4:** Physical and chemical properties of the sea buckthorn lipids.

Physical and Chemical Properties	Oil	
	Fruit Pulp	Seeds
Density at 20 °C, kg/m^3^	913.7	959.0
Acid number, mg KOH/g	1.5	6.5
Iodine number, mg J_2_/100 g	74.9	155.0
Unsaponifiable substances	3.23	1.85
Carotenoid content, mg/100 g	320.0 ± 3.0 ^a^	11.1 ± 0.05 ^a^
Content of tocopherols, mg/100 g	90.0 ± 2.0 ^a^	160.0 ± 0.1 ^a^
Fatty acid content, %		
saturated	32.8	13.4
oleic	50.6	16.4
linoleic	15.6	47.6
linolenic	-	18.4

^a^—Means in the same line for all or all types of oil followed by different letters are significantly different (*p* ≤ 0.05). Values are mean ± SD (*n* = 3).

**Table 5:** Fractional composition of the phospholipids under study.

Phospholipid Fractions	Content, %	
	Sunflower Phospholipids (SFP)	Soy Phospholipids (SBP)
Phosphatidylcholine	45.5 ± 2.5 ^b^	48.5 ± 2.5 ^d^
Lysophosphatidylcholine	1.0 ± 0.5 ^b^	-
Phosphatidylinositols	12.5 ± 2.7 ^a^	4.5 ± 1.2 ^d^
Phosphatidylserines	14.0 ± 1.3 ^a^	13.6 ± 2.4 ^c^
Phosphatidylethanolamine	14.5 ± 2.5 ^a^	9.8 ± 1.0 ^d^
Phosphatidic acids	12.5 ± 1.3 ^b^	23.6 ± 2.3 ^a^

^a^, ^b^, ^c^, ^d^—Means in the same line for all all types of phospholipids followed by different letters are significantly different (*p* ≤ 0.05). Values are mean ± SD (*n* = 3). SD—Standard deviation.

**Table 6:** Fatty acid composition of the phospholipid fractions.

Phospholipid Group	Content of Individual Fatty Acids, %							
	Myristine	Palmitic	Stearic	ΣS*	Palmitoleic	Oleic	Linoleic	ΣUS**
**Sunflower Phospholipids**								
Phosphatidylcholines	No	11.45	6.34	17.79	1.10	16.41	64.70	82.21
Phosphatidylinositols	Traces	23.51	7.34	30.85	1.50	15.42	52.23	69.15
Phosphatidylserines	No	12.62	7.12	19.74	0.22	15.00	65.04	80.26
Phosphatidylethanolamines	Traces	25.45	5.90	31.35	1.31	18.32	49.02	68.65
Phosphatidic acids	No	19.01	7.34	26.35	1.90	18.14	53.61	73.65
Diphosphatidylglycerols	No	20.01	8.50	28.51	0.59	16.22	54.68	71.49
Polyphosphatid acids	No	16.26	6.84	23.10	1.50	16.81	58.60	76.90
**Soy phospholipids**								
Phosphatidylcholines	No	17.91	5.70	23.61	19.30	49.39	7.70	76.39
Phosphatidylinositols	0.30	27.85	6.80	34.35	18.70	40.35	6.00	65.05
Phosphatidylserines	0.30	28.52	8.53	37.35	14.30	42.20	6.15	62.65
Phosphatidylethanolamines	No	18.50	6.40	24.90	23.97	45.18	5.95	75.10
Phosphatidic acids	No	26.40	5.15	31.55	18.81	43.34	6.30	58.45
Diphosphatidylglycerols	No	27.05	5.17	32.22	16.28	4535	6.15	67.78
Polyphosphatid acids	No	22.17	6.05	28.22	21.50	43.43	6.85	71.78

Σs*: total saturated fatty acids. ΣUS**: total unsaturated fatty acids.

**Table 7:** Colloidal stability of the emulsions.

Emulsion Sample Number	Emulsifier Mass Fraction, %		Emulsifier Hydrophilic-Lipophilic Balance	Intact Emulsion, %
	Hydrolyzed Soy Phospholipids	Fractionated Soybean Phospholipids		
1	1	- *	7	94.5
2	- **	1	3	98.5
3	0.5	0.5	5	96.0
5	0.6	0.4	5.4	95.5
6	0.4	0.6	4.6	99.5

* the component is absent in the sample; ** the component is absent in the sample.

**Table 8:** Quality factors.

Factor	Value
Mass fraction of lipids, %	47.0 ± 0.5
Mass fraction of moisture, %,	52.0 ± 0.5
Stability of emulsion, % of intact substance	99.0 ± 0.5
Hydrogen index (pH) at 20 °C	4.2 ± 0.2
Viscosity at 20 °C, mm^2^/s	36.2 ± 0.05
Peroxide number of the emulsion fat phase, mEqO_2_/kg	1.5 ± 0.2
The acid number of the emulsion fat phase, mg KOH/100 g	0.55 ± 0.05
Carotenoid content, mg/100 g	128
Tocopherol content, mg/100 g	64
Phospholipid content, mg/100 g	1500

## Data Availability

The data presented in this study are available on request from the corresponding author. The data are not publicly available due to the mandatory period before obtaining a patent for a developed functional product.
